# Evaluation of the most popular annual flowers sold in the United States and Europe indicates low visitation rates by pollinators and large variation among cultivars

**DOI:** 10.1093/jee/toae084

**Published:** 2024-05-13

**Authors:** David Smitley, Colin Oneil, Erica Hotchkiss, Erik Runkle, Jared Studyvin

**Affiliations:** Department of Entomology, Michigan State University, East Lansing, MI 48825-1115, USA; Department of Entomology, Michigan State University, East Lansing, MI 48825-1115, USA; Department of Entomology, Michigan State University, East Lansing, MI 48825-1115, USA; Department of Horticulture, Michigan State University, East Lansing, MI 48825-1115, USA; Department of Mathematics and Statistics, University of Wyoming, 3036, 1000 E. University Avenue, Laramie, WY 82071-3036, USA

**Keywords:** annual flower, pollinator, honey bee, bumble bee, syrphid fly

## Abstract

To better understand how frequently pollinators visit the most popular annuals and the variation among cultivars, we evaluated 3–6 cultivars, each of petunia, impatiens, begonia, geranium, pansy, and New Guinea impatiens. These 6 annuals account for 46.6% of all garden center annual flower sales in the United States. Flower visits by honey bees, bumble bees, syrphids, other Diptera and other Hymenoptera, combined, varied 3 to 10-fold among cultivars within each of the 6 popular annuals. Begonia and impatiens were visited more frequently by pollinators than pansy, petunia, NG impatiens, and geranium. The 4 most visited cultivars, begonia ‘Cocktail Brandy’, begonia ‘Ambassador Rose Blush’, impatiens ‘Accent Coral’, and impatiens ‘Super Elfin XP White’ attracted as many pollinators as a benchmark annual, marigold ‘Alumia Vanilla Cream’, considered as moderately attractive to pollinators. Some conclusions from this research may be helpful for homeowners, landscapers, growers, and breeders. First, the most popular annual flowers are not a good choice for the purpose of attracting and supporting pollinators. However, the large variation among cultivars provides an opportunity to select cultivars that are more attractive to pollinators, particularly for begonia and impatiens. If the most pollinator-visited cultivars of begonia and impatiens are labeled and promoted as such, it would be beneficial to pollinators in urban and suburban landscapes in the USA and Europe, where they comprise 10%–20% of all annual flowers purchased from garden centers.

## Introduction

Annual flowers account for more than half of all herbaceous plants purchased at garden centers in the United States, with a total annual sale value of $1.97 billion (USDA-NASS 2020). When the 52 types of annual flowers listed in the most recent USDA survey are sorted by the value of annual sales, the top 6 are petunia (*Petunia X hybrida*, $238.1 million), geranium (*Pelargonium zonale*, $217.3 million), pansy (*Viola X wittrockiana*, $156.6 million), begonia (*Begonia semperflorens,* $133.7 million), impatiens (*Impatiens walleriana*, $91.1 million), and New Guinea impatiens (*Impatiens hawkeri*, $81.7 million). The total sales value for these 6 annual flowers is $918.5 million, 46.6% of the value of all annual flowers, and roughly equal to the value of all herbaceous perennials sold in the USA each year (USDA-NASS 2020). Therefore, close to half of all the annual flowers planted each year in the United States consists of these 6 flowers, yet little is published about their attraction or lack of attraction to pollinators. The only information available is their omission or rare mention in visitation data from observations made in gardens or at garden centers (Comba et al. 1999, Garbuzov and Ratnieks 2014a,b, Lindter 2014, Shackleton and Ratnieks 2015, Sikora et al. 2016, Garbuzov et al. 2017, Rollings and Goulson 2019).

A growing body of evidence is leading to consensus that pollinators are facing a worldwide decline in both wild and managed systems due to loss of habitat, parasites, diseases, and multiple exposures to pesticides (Potts et al. 2010, Ollerton et al. 2014, Goulson et al. 2015). In urban areas, bees can be exposed to harmful levels of neonicotinoid insecticides when pollen from flowers recently grown in nurseries is collected (Lentola et al. 2017). Analysis of pollen brought to hives by honeybee colonies located near commercial nurseries reveals that neonicotinoid concentrations in pollen are mostly in the subacute but potentially harmful range of 2–6 ppb (Stoner et al. 2019, Cecala and Wilson Rankin 2021).

As public awareness of this problem increases, more gardeners and landscapers want to participate in pollinator conservation (Toomey and Domroese 2013, Campbell et al. 2017, Wignall et al. 2019). This has been documented in surveys of garden center consumers. Pollinator-friendly plants, or plants labeled in this way, tend to be preferred by consumers and may support a higher price (Khachatryan et al. 2017, Khachatryan and Rihn 2018, Wei et al. 2021). Unfortunately, plants may be marketed as pollinator-friendly when they are not very attractive to pollinators or have been treated with insecticides (Garbuzov and Ratnieks, 2014b, Garbuzov et al. 2017, Lentola et al. 2017). Part of the problem is a lack of knowledge about pollinator attraction to intensively bred and selected cultivars of ornamental plants. Selection is based heavily on vigor, growth habit, flower color, and flower abundance (Hicks et al. 2002, Anderson 2007, Guo and Warner 2020). Many cultivars of annual flowers have reduced floral rewards or poor access by pollinators compared with the near-original flower source (Comba et al. 1999, White 2016). This happens because cultivar selection tends to favor genotypes that are diverting plant resources to showy flowers, making fewer resources available for nectar and pollen (Shibuya et al. 2013).

Pollinator attraction to annuals and perennials grown in nurseries for garden centers may vary considerably among cultivars (Yeargan and Colvin 2009, Nabors et al. 2022). For example, when Garbuzov and Ratnieks (2014a) evaluated the relative attractiveness of 13 cultivars of lavender (*Lavandula*), the most visited cultivar outperformed the least visited cultivar by over 100-fold. This makes it difficult to recommend garden center flowers that are attractive to pollinators unless cultivar names are included. Large variation in attraction to pollinators among cultivars within a species or horticultural cross like *Petunia X hybrida* becomes problematic when many cultivars are sold at garden centers. One of the largest growers of annual flowers in the United States is currently offering 65 cultivars of petunias in its online catalog (Proven Winners 2024). Another part of the world where large numbers of annual flowers are sold in flats and pots at garden centers is Europe (AIPH 2021). Plant production statistics for the United Kingdom in 2021 rank the top 7 most popular annuals in Europe, in order from most to least number grown, as petunia, pansy, impatiens, begonia, marigold, geranium, and New Guinea impatiens (AIPH 2021). All the top 6 annuals that we evaluated are among their top 7. One difference is that Marigold is number 5 in the United Kingdom but not among the top 7 in the United States.

To provide better information about the most popular annual flowers grown in the United States and Europe, we evaluated 3–5 cultivars, each, of petunia, geranium, pansy, begonia, impatiens, and New Guinea impatiens for their attraction to honey bees, wild bees, bumble bees, and syrphids. One representative cultivar for each of 2 annuals, zinnia ‘Zahara Sunburst’ and marigold ‘Alumia Vanilla Cream’ (Erickson et al. 2020, Browning et al. 2023), and 2 perennials catmint ‘Walker’s Low’, and coneflower ‘Pow Wow Wild Berry’ (White 2016, Anderson et al. 2022), reported as attractive to pollinators, were evaluated in adjacent replicated blocks as benchmark plants. Our hypothesis, based on previous nonreplicated observations of pollinator visits to flowers in garden centers, is that cultivars within each genus type of the most popular annual flowers vary considerably in their attraction to pollinators and attract fewer pollinator visits than marigold and zinnia.

## Materials and Methods

Research plots for both years were located at the Michigan State University Horticulture Teaching and Research farm. The plot area is located on the north edge of the research farm, bordered by an abandoned wet field with a wide variety of early colonizing grasses, broadleaf weeds, and small shrubs on one side. South and east of our plot area are intensely managed horticultural crops, including some that are treated with insecticide. No insecticides were applied within 100 m of our research plots.

**Fig. 1.**
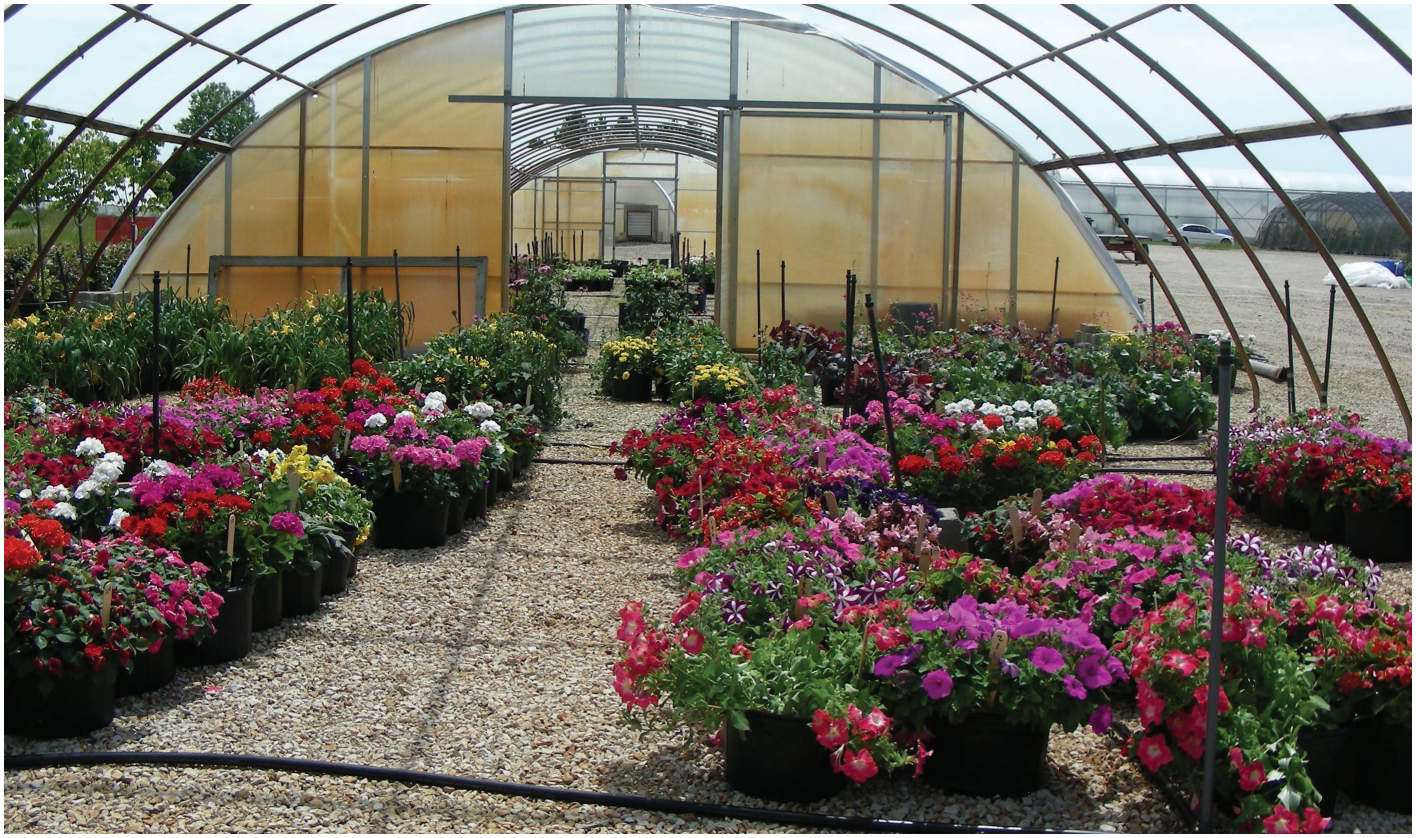
Popular annuals at Michigan State University Horticulture Farm in June 2017. Three of the 6 replicate blocks are visible. Each block consists of 2 rows of annuals, with one cultivar per container. All 25 cultivars are randomly placed into each block for a total of 150 containers.

**Fig. 2.**
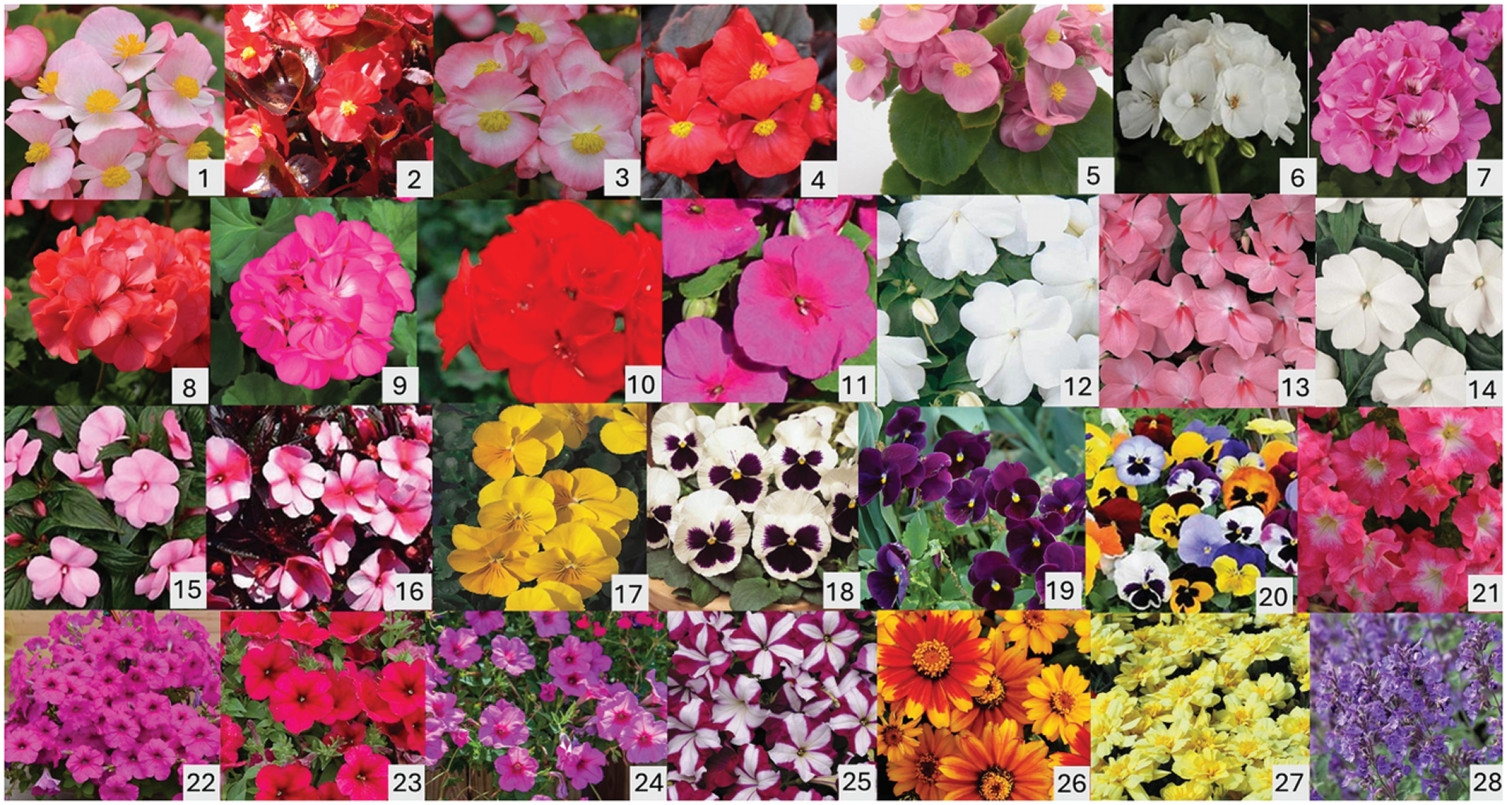
Cultivars of popular annual flowers evaluated in field plots in 2017 and 2018, numbered and in order as they are listed in Table 1.

In 2017, 5 cultivars each of wax begonia (*Begonia semperflorens*), petunia (*Petunia* × *hybrida*), and geranium (*Pelargonium zonale*), 4 cultivars of pansy (*Viola* × *wittrockiana*), and 3 cultivars each of impatiens (*Impatiens walleriana*) and New Guinea impatiens (*Impatiens hawkeri*) were evaluated throughout most of the growing season for attraction to pollinators (Table 1; Figs. 1 and 2). Each cultivar is one treatment for a total of 25 treatments. Each treatment was replicated 6 times for a total of 150 plants in individual containers. One replicate of each treatment was randomly placed into each of 6 blocks. Each block consisted of a line of plants in containers, with 0.1–0.2 m between containers so that the foliage barely overlapped with the next plant. In addition, in order evaluate pollinator activity in the area, 2 annual flowers, zinnia (*Zinnia elegans* ‘Zahara Sunburst’) and marigold (*Tagetes patula* ‘Alumia Vanilla Cream’) and 2 perennial flowers, catmint (*Nepeta X faassenii* ‘Walker’s Low’) and coneflower (*Echinacea purpurea* ‘Pow Wow Wild Berry’), all of which are known to be attractive to pollinators, were evaluated in adjacent replicated plots, located 5 m away from our main plots (White 2016, Rollings and Goulson 2019, Erickson et al. 2020, Anderson et al. 2022).

**Table 1.** Common and scientific names for cultivars of begonia, geranium, impatiens, New Guinea impatiens, pansy, and petunia were evaluated for pollinator visits at the Michigan State University Horticulture Farm in 2017 and 2018. Numbers in parentheses following cultivar names correspond to appearance in Fig. 2. Written referrals in the text to annual flower genus types in this table appear as the common name in the first column, while referrals to cultivar names appear as the common name followed by cultivar

Annual flower	Species or cross	Cultivar
Begonia	*Begonia sempervirens*	‘Cocktail Brandy’ (1)
		‘Bada Boom Scarlet’ (2)
		‘Ambassador Rose Blush’ (3)
		‘Senator Scarlet’ (4)
		‘Super Olympia Rose’ (5)
Geranium	*Pelargonium zonale*	‘Tango White’ (6)
		‘Tango Deep Pink’ (7)
		‘Americana Coral’ (8)
		‘Rocky Mountain Deep Rose’ (9)
		‘Tango Dark Red’ (10)
Impatiens	*Impatiens walleriana*	‘Super Elfin Ruby’ (11)
		‘Super Elfin XP White’ (12)
		‘Accent Coral’ (13)
New Guinea impatiens	*Impatiens hawker*	‘Divine White’ (14)
(NG impatiens)		‘Sonic Light Pink’ (15)
		‘Super Sonic Sweet Cherry’ (16)
Pansy	*Viola* × *wittrockiana*	‘Yellow’ (17)
		‘White Blotch’ (18)
		‘Matrix Purple’ (19)
		‘Mix’ (20)
Petunia	*Petunia* × *hybrida*	‘Dreams Coral Morn’ (21)
		‘Easy Wave Neon Rose’ (22)
		‘Easy Wave Berry Velour’ (23)
		‘Easy Wave Pink’ (24)
		‘Easy Wave Burgundy Star’ (25)
**Annual standard**	**Species or cross**	**Cultivar**
Zinnia	*Zinnia elegans*	‘Zahara Sunburst’ (26)
Marigold	*Tagetes patula*	‘Alumia Vanilla Cream’ (27)
**Perennial standard**	**Species or cross**	**Cultivar**
Catmint	*Nepeta* × *faassenii*	‘Walker’s Low’ (28)
Coneflower	*Echinacea purpurea*	‘Pow Wow Wild Berry’

All 25 cultivars in this experiment are popular cultivars sold at garden centers in the United States. Every effort was made to grow plants in the greenhouse in the same way they are produced commercially for spring sale at garden centers. Production practices like pinching flower buds and cold treatments were scheduled to have large plants in full bloom in early June. All plants were grown from seeds or plugs in the Michigan State University Plant and Soil Science Greenhouses starting in February or March. Plants received 9 h of light per day from high-pressure sodium lamps and were kept at 20 °C. Irrigation water was premixed with 125 ppm Orchid RO Water Special from Greencare Fertilizers, INC (Kankakee, IL, USA). No pesticides were used. Biological control was used for pest management, beginning with preventive releases of a predator mite, *Neoseiulus cucumeris* (Oudemans), applied at the recommended rate for thrips control.

When plants reached an appropriate height, they were transplanted into 18.9 L plastic containers (4 individual plants per container). All plants were in excellent condition, filling the 18.9 L containers with attractive foliage covered by a solid layer of open flowers. Replicates of each cultivar appeared nearly identical, with no noticeable differences in plant size or flower production. Only one plant was not in full bloom; one of the 4 benchmark plants, coneflower ‘Pow Wow Wild Berry’, due to the late arrival of those starter plugs in 2017. By the first week of June in 2017 and 2018, all 174 test plants were fully grown and ready to be moved to the MSU Horticulture Farm. All plants were fertilized with 38.3 g per container of Ozmacote (Marysville, OH, USA) on 1st June, then moved outdoors to the research plot area.

All annuals were healthy and in full bloom when moved outdoors to the horticulture farm in both years. Cultivars within each type of annual flower differed from each other primarily in flower color or in patterns of color (Figs. 1 and 2). By late June, new growth filled gaps between containers so that each row of one replicate of 25 cultivars was a solid row of flowers without any overgrowth of neighboring plants. All cultivars except for coneflower ‘Pow Wow Wild Berry’ were in full bloom throughout June, July, and August. Coneflower ‘Pow Wow Wild Berry’ plants remained small, with few flowers throughout the season. The potted flowers in our test received full sun and overhead irrigation for 20 min each day. We also hand-watered individual containers when plants appeared to be too dry. Plants were deadheaded and cleared of spiderwebs weekly. To supplement natural pollinators in the area, 3 hives of managed honey bees, *Apis mellifera* L., and 3 hives of bumble bees, *Bombus impatiens* Cresson, were placed in a tree-shaded area about 20 m from the blocks. Honey bee hives were provided by Zachary Huang (MSU), and bumble bee hives came from Biobest. Inc (Leamington, Ontario, Canada).

In 2018, plant rearing was repeated as previously described with the following exception: in the 1st week of August, impatiens and New Guinea Impatiens were covered with shade cloth placed 0.3 m above the plants to prevent them from dropping leaves due to sun-scald. The randomized complete block design with the same 25 cultivars was repeated for a second year of evaluation in 2018.

### Pollinator Sampling

All pollinators observed landing on any flower of annuals growing in one container were collected continuously during a 1-min sample period, 1–3 times/week from early June to late August. Collections were only made under favorable conditions for pollinator foraging (between 15.6° C and 32.2 °C, no precipitation or dew on flowers, and winds <24 kph). Pollinators were collected using 18.0 V insect vacuums from Bioquip (Rancho Domingo, CA, USA). All collected insects were euthanized with ethyl acetate, placed into labeled scintillation vials and transported in a cooler for storage at 4 °C. All insects were pinned and labeled the same week they were collected.

One to ten specimens of each morphotype of wild bee were sent to Jason Gibbs, Curator of the J.B. Wallis and R.E. Roughley Museum of Entomology (Winnipeg, Manitoba, Canada) for identification. After the identified bees were returned to us, wild bees were identified as species by comparison to bees identified by Jason Gibbs. This was not possible for half of the individuals in the genera *Lasioglossum* (Halictidae) and *Melissodes* (Apidae), which were only identified to genus because some species were too similar for us to separate. Syrphid fly morphotypes were identified as species by Gary Parsons, Emeritus Curator of the Michigan State University, using Skevington et al. (2019) and comparisons with museum specimens. All identifications, other than for wild bees, were confirmed by Gary Parsons. Voucher specimens for each species were deposited in the MSU Arthropod Research Collection. In 2017, all plants were sampled 23 times from 7th June to 25th August. In 2018, they were sampled 19 times, from 11th June to 30th August, except for impatiens ‘Super Elfin Ruby’ and impatiens ‘Super Elfin XP White’ (sampled 14 times from 11th June to 8th August) and petunia ‘Easy Wave Berry Velour’ (sampled 15 times from 11th June to 9th August). Sampling for those 3 cultivars was discontinued after 9th August because fewer flowers were being produced.

### Statistical Analysis

All analyses were conducted in R Version 4.1.2 (R Core Team 2021). Pollinators collected from annual flowers in research plots were initially split into 5 pollinator groups: honey bees, bumble bees, syrphids, other Hymenoptera, and other Diptera. Low visitation rates for most of the cultivars being evaluated did not provide enough data to compare visitation rates for each group of pollinators. Instead, comparisons of mean visitation rates were made after combining the 5 groups of pollinators into an overall pollinator visitation rate. All insects collected from flowers that belong to one of these 5 groups were used in the data analysis for all pollinators. For each of the 150 flowering plants in our field plot (25 cultivars × 6 replicates), flower visitation data were summed across all sample periods and then divided by the number of sample periods. The results are presented as overall pollinator visits per minute for each cultivar in each year.

A Poisson regression approach was used to assess the differences in pollinator visitation rates among the cultivars of each annual (Bilder and Loughin 2014). All possible pairwise tests between the cultivars within each type of annual were done within the Poisson regression framework. The initial alpha level is 0.05. Tukey’s method was used to correct for compounding of the alpha level when conducting multiple dependent tests (Kuehl 2000). This analysis was done for each year separately, with pollinator visitation counts set as the response variable and the time of sampling as the dependent variable. A Tukey-like pairwise comparison of each cultivar was used to further assess differences. Ratios, rather than differences, were utilized due to the log-transformed nature of modeled Poisson-count outcomes. In the case a ratio was less than one, its reciprocal was reported instead; this way, all calculated ratios were greater than one. Pollinator visits to cultivars within a type of annual flower were considered different from each other if the pairwise ratio was significantly (*P* = 0.05) greater than 1.0. In addition to comparing cultivars within a type of flower to each other, we also compared pollinator visits to the 6 types of annual flowers in our field trial (begonia, geranium, impatiens, NG impatiens, pansy, and petunia) to each other, using cultivars as replications. Mean pollinator visitation rates were compared with Tukey’s HSD at *P* = 0.05.

## Results

Throughout 2017 and 2018, more than 99% of all flower visitors were members of the Hymenoptera or Diptera. Honey bees were frequent flower visitors (*n* = 250 for both years combined), followed by syrphids (227), other Diptera (202), bumble bees (121), and other Hymenoptera (118). Five species of syrphids were collected, with *Toxomerus marginatus* being the dominant species, accounting for 86% of all syrphids (Table 2). Thirteen species of ‘other Hymenoptera’ were collected, with sweat bees accounting for 62% and wasps accounting for 29% of all individuals collected. Flies in the Anthomyiidae were the largest group of ‘other Diptera’ (52% of all collected), followed by Muscidae (27%).

**Table 2.** Pollinator groups used in data analysis comparing cultivars of popular annual flowers: ‘honey bees’, ‘bumble bees’, ‘syrphids’, ‘Other Hymenoptera’, and ‘Other Diptera’. Classification and number of individuals collected in each pollinator category are shown. Data are for insects collected from the flowers of all cultivars of annual flowers from June to August of 2017 and 2018

Pollinator group	Family	Genus	Species	Collected (*n*)
Honey bee	Apidae	*Apis*	*mellifera*	250
Bumble bee	Apidae	*Bombus*	*impatiens*	121
Syrphids	Syrphidae (syrphid flies)	*Toxomerus*	*marginatus*	196
		*Syrphus*	*ribesii*	16
		*Eristalis*	*tenax*	12
		*Eristalis*	*transversa*	2
		*Eristalis*	*arbustorum*	1
Other Hymenoptera	Sweat bees (Halictidae)	*Lasioglossum*	*admirandum*	17
		*Lasioglossum*	*laevissimum*	1
		*Lasioglossum*	*lineatulum*	1
		*Lasioglossum*	*paradmirandum*	1
		*Lasioglossum*	*pilosum*	1
		*Lasioglossum*	*leucozonium*	1
		*Lasioglossum*	Undetermined	38
		*Halictus*	*confusus*	10
		*Halictus*	Undetermined	3
	Plasterer bees (Colletidae)	*Hylaeus*	*affinus*	4
		*Hylaeus*	*modestus*	1
		*Hylaeus*	*leptocephalus*	1
		*Hylaeus*	*mesillae*	1
	Apidae (carpenter bees)	*Ceratina*	*calcarata*	1
		*Xylocopa*	*virginica*	3
	Wasps (Apocrita) (all families)	Undetermined	Undetermined	34
Other Diptera	Anthomyiidae	Undetermined	Undetermined	105
	Muscidae	Undetermined	Undetermined	54
	All other families	Undetermined	Undetermined	43

### Pollinator Visits in 2017

**Fig. 3.**
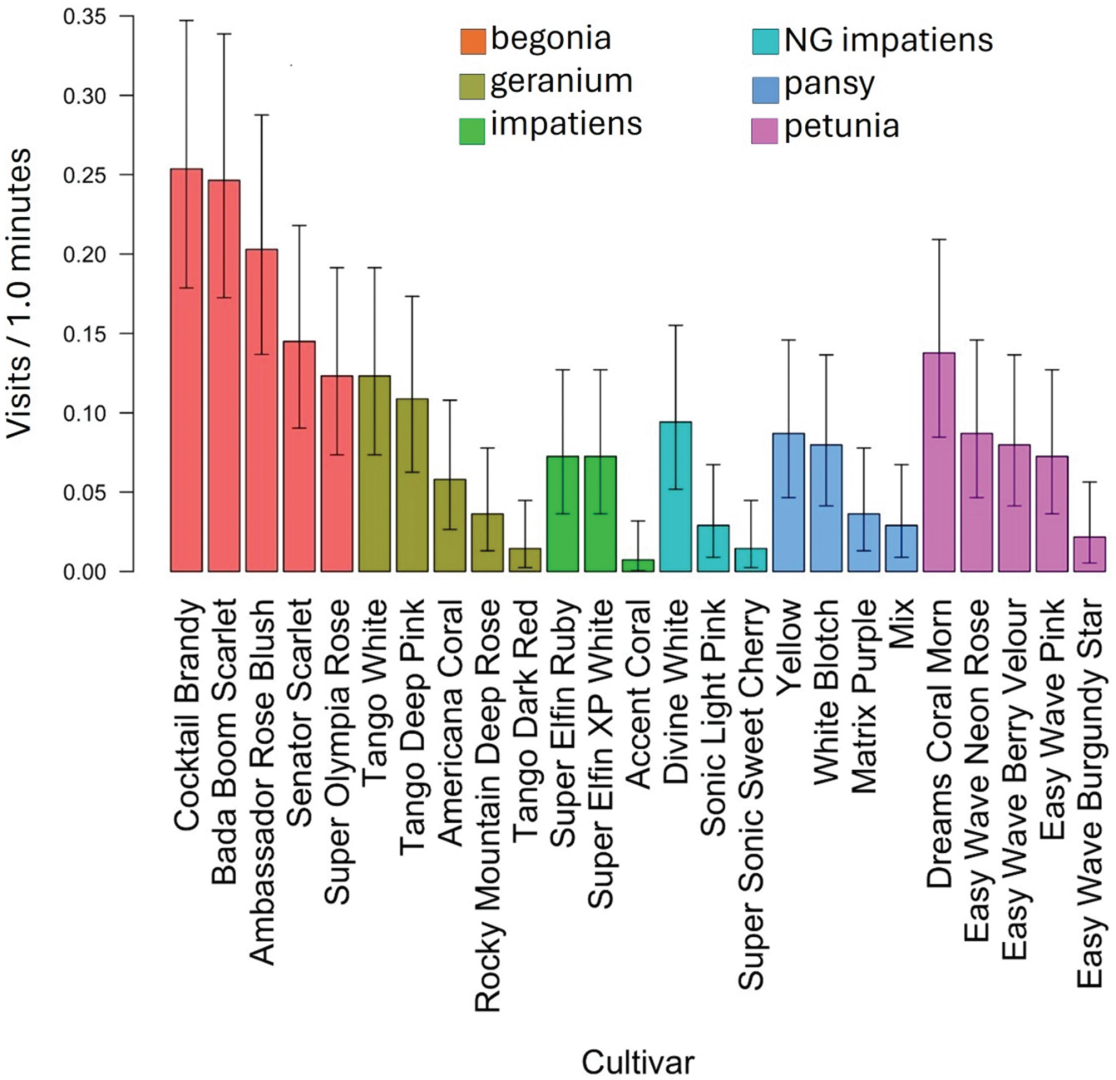
Mean pollinator visitation rate (visits/min) for the entire 2017 growing season for all pollinator groups combined for cultivars of begonia, geranium, impatiens, New Guinea impatiens, pansy, and petunia. Results for each of the 25 cultivars are grouped by flower type and put in order within each flower type from most visited to least visited. Comparisons among flower types with cultivars as replications are shown in Fig. 4. Error bars indicate a 95% confidence interval about means.

Visitation rates for all pollinators combined were compared among cultivars within each type of annual flower. For begonia, pansy, impatiens, and New Guinea impatiens, there were no significant differences among cultivars (Fig. 3). However, geranium and petunia did have cultivars that varied in attraction. Geranium cultivars ‘Tango White’ and ‘Tango Deep Pink’ had the highest visitation rates, each with 6-fold more pollinator visits than the lowest ranked cultivar, ‘Tango Dark Red’ (Fig. 3; Table 3, *P* = 0.015 and 0.028, respectively). There were no significant differences among other cultivars. For petunia, the highest ranked cultivar, ‘Dreams Coral Morn’, had 6-fold more visits from pollinators than ‘Easy Wave Burgundy Star’, the lowest ranked cultivar (Fig. 3; Table 3, *P* = 0.014). There were no differences among the remaining cultivars of petunia.

**Table 3.** 2017 statistics for Tukey-like pairwise comparison of cultivars within each type of annual flower. Pollinator visitation to each of the 2 cultivars in each pair below is considered different from each other if the *P*-value is <0.05. *P*-values <0.05 are indicated by bold print

Cultivar pairs	Flower type	b0	Lo 2.5	Hi 97.5	C alpha	*P*-value
Super Olympia Rose/Cocktail Brandy	Begonia	2.058	0.856	4.951	2.723	0.163
Super Olympia Rose/Bada Boom Scarlet	Begonia	2.000	0.828	4.830	2.723	0.201
Senator Scarlet/Cocktail Brandy	Begonia	1.750	0.762	4.021	2.723	0.353
Senator Scarlet/Bada Boom Scarlet	Begonia	1.700	0.737	3.924	2.723	0.414
Super Olympia Rose/Ambassador Rose Bl.	Begonia	1.647	0.661	4.103	2.723	0.567
Senator Scarlet/Ambassador Rose Blush	Begonia	1.400	0.587	3.338	2.723	0.828
Cocktail Brandy/Ambassador Rose Blush	Begonia	1.250	0.589	2.653	2.723	0.928
Bada Boom Scarlet/Ambassador Rose Bl.	Begonia	1.214	0.569	2.590	2.723	0.957
Super Olympia Rose/Senator Scarlet	Begonia	1.176	0.442	3.132	2.723	0.991
Cocktail Brandy/Bada Boom Scarlet	Begonia	1.029	0.504	2.104	2.723	1.000
Tango White/Tango Dark Red	Geranium	8.500	1.328	54.386	2.696	**0.015**
Tango Deep Pink/Tango Dark Red	Geranium	7.500	1.157	48.615	2.696	**0.028**
Tango Dark Red/Americana Coral	Geranium	4.000	0.562	28.479	2.696	0.299
Tango White/Rocky Mountain Deep Rose	Geranium	3.400	0.961	12.024	2.696	0.063
Tango Deep Pink/Rocky Mountain Deep R.	Geranium	3.000	0.832	10.81	2.696	0.132
Tango Dark Red/Rocky Mountain Deep R.	Geranium	2.500	0.313	19.95	2.696	0.745
Tango White/Americana Coral	Geranium	2.125	0.733	6.161	2.696	0.297
Tango Deep Pink/Americana Coral	Geranium	1.875	0.632	5.560	2.696	0.506
Rocky Mountain Deep Rose/Americana C.	Geranium	1.600	0.388	6.589	2.696	0.892
Tango White/Tango Deep Pink	Geranium	1.133	0.470	2.731	2.696	0.995
Super Elfin Ruby/Accent Coral	Impatiens	10.000	0.390	256.12	2.305	0.216
Super Elfin XP White/Accent Coral	Impatiens	10.000	0.390	256.12	2.305	0.216
Super Elfin XP White/Super Elfin Ruby	Impatiens	1.000	0.251	3.986	2.305	1.000
Super Sonic Sweet Cherry/Divine White	NG Impatiens	6.500	0.955	44.261	2.326	0.058
Sonic Light Pink/Divine White	NG Impatiens	3.250	0.767	13.773	2.326	0.135
Super Sonic Sweet Cherry/Sonic Light Pink	NG Impatiens	2.000	0.224	17.821	2.326	0.736
Yellow/Mix	Pansy	3.000	0.644	13.967	2.557	0.255
White Blotch/Mix	Pansy	2.750	0.581	13.027	2.557	0.337
Yellow/Matrix Purple	Pansy	2.400	0.581	9.909	2.557	0.384
White Blotch/Matrix Purple	Pansy	2.200	0.523	9.256	2.557	0.491
Mix/Matrix Purple	Pansy	1.250	0.209	7.465	2.557	0.988
Yellow/White Blotch	Pansy	1.091	0.359	3.317	2.557	0.997
Easy Wave Burgundy Star/Dreams Coral M.	Petunia	6.333	1.279	31.350	2.705	**0.014**
Easy Wave Neon Rose/Easy Wave Burg. St.	Petunia	4.000	0.759	21.075	2.705	0.152
Easy Wave Burg. Star/Easy Wave Berry Vel.	Petunia	3.667	0.686	19.611	2.705	0.212
Easy Wave Pink/Easy Wave Burgundy Star	Petunia	3.333	0.612	18.150	2.705	0.295
Easy Wave Pink/Dreams Coral Morn	Petunia	1.900	0.695	5.195	2.705	0.406
Easy Wave Berry Velour/Dreams Coral Mor.	Petunia	1.727	0.651	4.581	2.705	0.540
Easy Wave Neon Rose/Dreams Coral Morn	Petunia	1.583	0.613	4.091	2.705	0.675
Easy Wave Pink/Easy Wave Neon Rose	Petunia	1.200	0.399	3.613	2.705	0.991
Easy Wave Pink/Easy Wave Berry Velour	Petunia	1.100	0.357	3.388	2.705	0.999
Easy Wave Neon Rose/Easy Wave Berry Ve.	Petunia	1.091	0.372	3.195	2.705	0.999

**Fig. 4.**
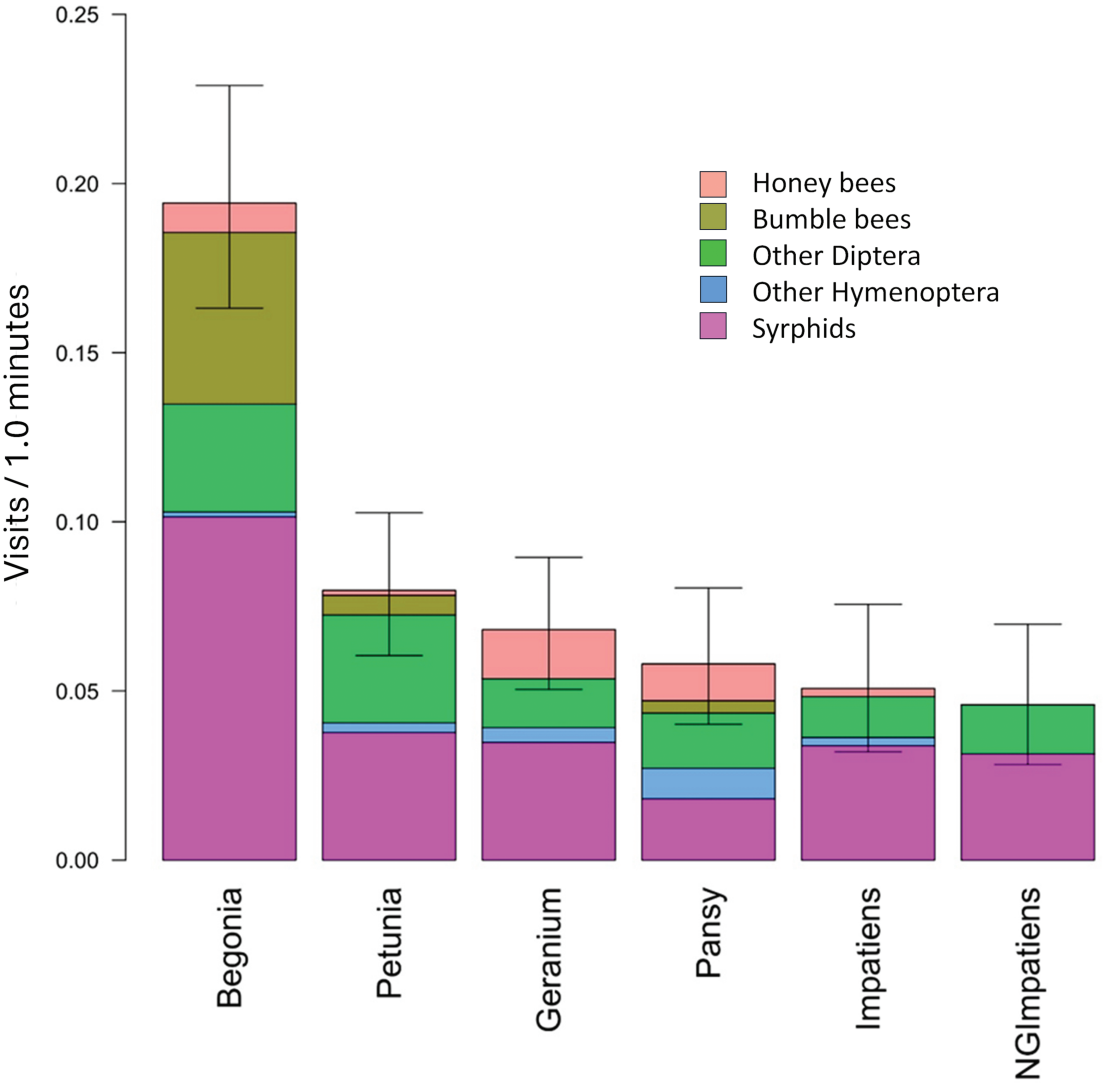
In 2017, a comparison of pollinator visits to begonia, petunia, geranium, pansy, impatiens, and New Guinea impatiens, with cultivars of each as replicates. Each bar is subdivided into visits per minute for honey bees, bumble bees, Other Diptera, Other Hymenoptera, and syrphids. Error bars at the top of each combined bar are 95% confidence limits about the mean for all 5 groups of pollinators combined. Cultivars of each flower type used as replicates are shown in Fig. 3.

In 2017, when total pollinator visits to begonia, geranium, impatiens, NG impatiens, pansy, and petunia were compared with each other using cultivars as replicates, begonia had more visits (0.19/min) than all other flower types (*F* = 7.20, *df* = 5,19, *P* < 0.001, Fig. 4). There were no differences among geranium, impatiens, NG impatiens, pansy, and petunia, all of which had less than 0.08 pollinator visits/min (Table 3). The mean ± SD number of visits per minute by our 5 pollinator groups to all annuals in our research plots with types of annuals as replicates, listed from most to least visits, are syrphids (0.042 ± 0.012), Other Diptera (0.024 ± 0.006), bumble bees (0.010 ± 0.008), honey bees (0.006 ± 0.0025), and Other Hymenoptera (0.003 ± 0.0012) (Fig. 4). In 2017, 71.1% of Other Hymenoptera were solitary bees (Table 2). Bumble bees heavily favored begonia, accounting for 83.3% of bumble visits to all flower types (Fig. 4). Honey bee visits were mostly to geranium (0.014/min), pansy (0.01/min) and begonia (0.008/min), while petunia, impatiens, and NG impatiens all received less than 0.002 visits per min.

**Fig. 5.**
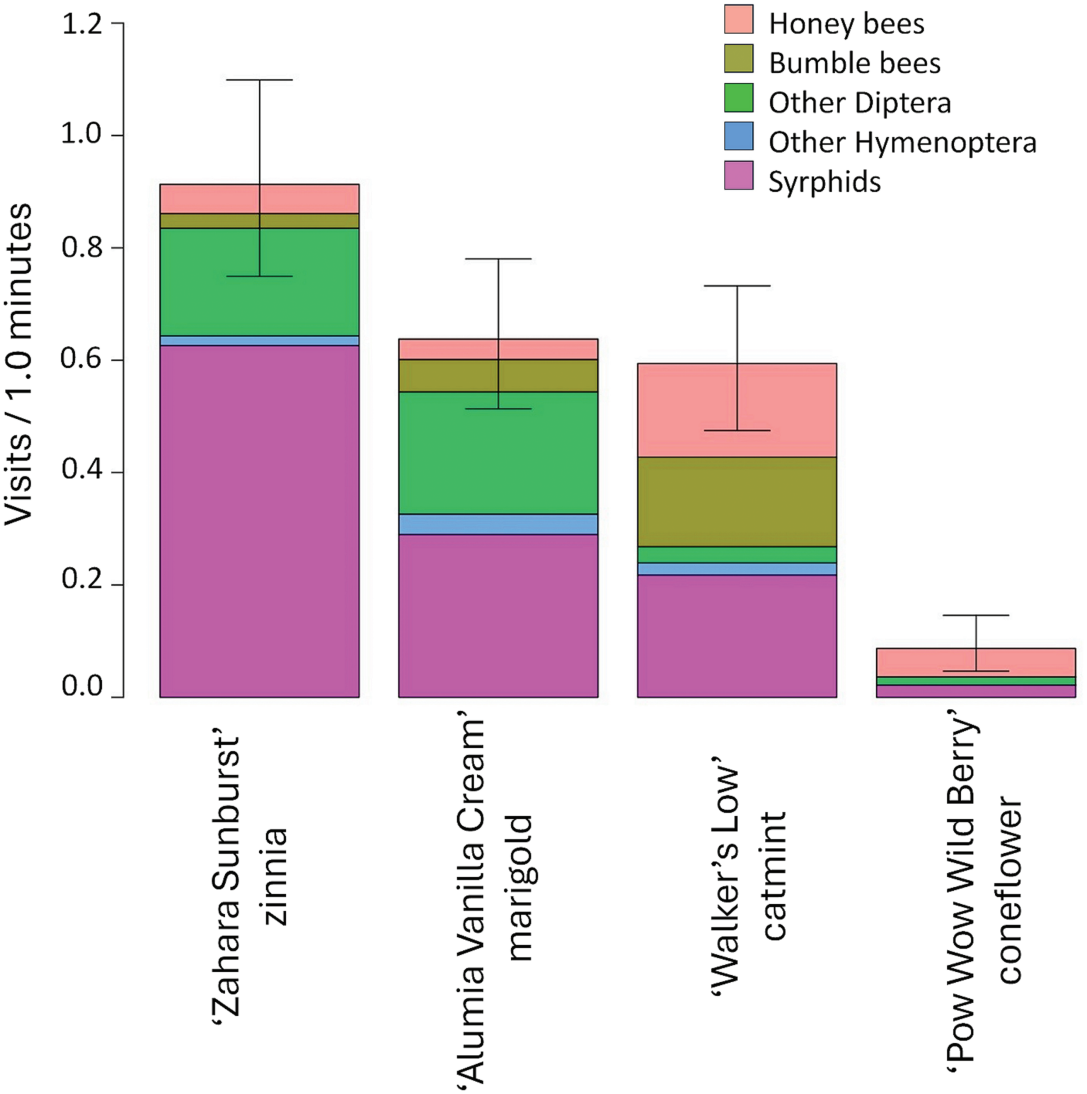
In 2017, results for pollinator-attractive benchmark plants. Data are the mean seasonal visitation rates to zinnia ‘Zahara Sunburst’, marigold ‘Alumia Vanilla Cream’, catmint ‘Walker’s Low’, and coneflower ‘Pow Wow Wild Berry’. The bar above each cultivar is subdivided into visits per minute for honey bees, bumble bees, syrphids, other Diptera, and other Hymenoptera. The error bars at the top of each combined bar are the 95% confidence limits for mean visits per minute for all 5 groups of pollinators combined.

In 2017, mean total pollinator visitation ± SD to adjacent replicated plots of our benchmark plants were 0.91 ± 0.14 for zinnia ‘Zahara Sunburst’, 0.63 ± 0.11 for marigold ‘Alumia Vanilla Cream’, 0.60 ± 0.11 for catmint ‘Walker’s Low’ and 0.095 ± 0.035 for coneflower ‘Pow Wow Berry’ (Fig. 5). ‘Pow Wow Wild Berry’ did not grow well due to the poor quality of the starter plants. The proportion of visits by each group of pollinators to the benchmark plants is similar to that for all annual flowers, with the most visits being made by syrphids, followed in decreasing order by Other Diptera, honey bees, bumble bees, and Other Hymenoptera. Catmint ‘Walker’s Low’ had more than 2-fold the number of honey bee and bumble visits (0.182 ± 0.042/min and 0.178 0.042/min, respectively) than the other benchmarks, while zinnia ‘Zahara Sunburst’ had more than 2-fold the number of syrphid visits (0.63 ± 0.098/min) than any other benchmark (Fig. 5).

### Pollinator Visits in 2018

**Fig. 6.**
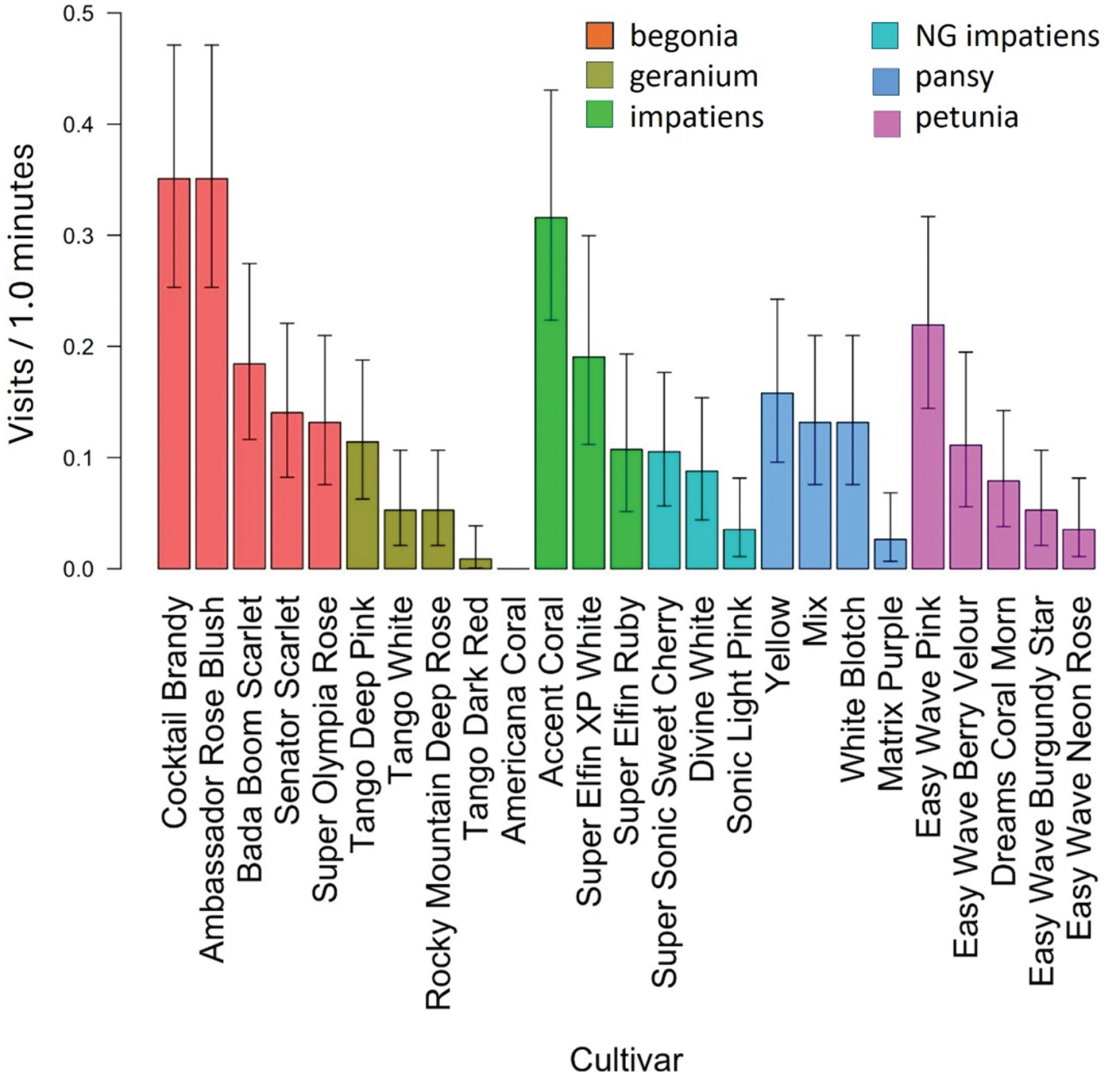
In 2018, mean pollinator visits per minute for the entire 2018 growing season for all pollinator groups combined. Results for each of the 25 cultivars are grouped by flower type and put in order within each flower type from most visited to least visited. Comparisons among flower types with cultivars as replications are shown in Fig. 7. Error bars indicate a 95% confidence limit about means.

In 2018, some cultivars within each of the 6 types of annual flowers, except for New Guinea impatines, were visited more than other cultivars of the same type. Begonia cultivars ‘Cocktail Brandy’ and ‘Ambassador Rose Blush’ had more pollinator visits than ‘Super Olympia Rose’ and ‘Senator Scarlet (Fig. 6; Table 4, *P* = 0.01 for all 4 comparisons). One cultivar of geranium, ‘Tango Deep Pink’, had more pollinator visits than geranium ‘Tango Dark Red’, (Fig. 6; Table 4, *P* = 0.05). Impatiens 'Accent Coral' had more visits than impatiens 'Super Elfin Ruby. For cultivars of pansy, ‘Yellow’ had more visits than ‘Matrix Purple’ (Fig. 6; Table 4, *P* = 0.01). For petunia, ‘Easy Wave Pink’ had more visits than ‘Easy Wave Neon Rose’ and ‘Easy Wave Burgundy Star’ (Fig. 6; Table 4, *P* = 0.028 and 0.05, respectively). No other cultivars within a type of annual flower were significantly different from each other.

**Table 4.** 2018 statistics for Tukey-like pairwise comparison of cultivars within each annual flower. Pollinator visitation to each of the 2 cultivars in each pair below is considered different from each other if *P* < 0.05. *P*-values <0.05 are indicated by bold print

Cultivar pairs	Flower type	b0	Lo 2.5	Hi 97.5	C alpha	*P*-value
Super Olympia Rose/Cocktail Brandy	Begonia	2.667	1.248	5.698	2.716	**0.004**
Super Olympia Rose/Ambassador Rose Blush	Begonia	2.667	1.248	5.698	2.716	**0.004**
Senator Scarlet/Cocktail Brandy	Begonia	2.500	1.191	5.250	2.716	**0.007**
Senator Scarlet/Ambassador Rose Blush	Begonia	2.500	1.191	5.250	2.716	**0.007**
Cocktail Brandy/Bada Boom Scarlet	Begonia	1.905	0.969	3.744	2.716	0.070
Bada Boom Scarlet/Ambassador Rose Blush	Begonia	1.905	0.969	3.744	2.716	0.070
Super Olympia Rose/Bada Boom Scarlet	Begonia	1.400	0.600	3.268	2.716	0.814
Senator Scarlet/Bada Boom Scarlet	Begonia	1.312	0.571	3.017	2.716	0.899
Super Olympia Rose/Senator Scarlet	Begonia	1.067	0.433	2.627	2.716	1.000
Cocktail Brandy/Ambassador Rose Blush	Begonia	1.000	0.571	1.752	2.716	1.000
Tango Deep Pink/Tango Dark Red	Geranium	13.000	0.978	172.8	2.604	0.053
Tango Dark Red/Rocky Mountain Deep Rose	Geranium	6.000	0.406	88.66	2.604	0.348
Tango White/Tango Dark Red	Geranium	6.000	0.406	88.66	2.604	0.348
Tango White/Tango Deep Pink	Geranium	2.167	0.633	7.417	2.604	**0.407**
Tango Deep Pink/Rocky Mountain Deep Rose	Geranium	2.167	0.633	7.417	2.604	**0.407**
Tango White/Rocky Mountain Deep Rose	Geranium	1.000	0.237	4.219	2.604	1.000
Super Elfin Ruby/Accent Coral	Impatiens	4.000	1.058	15.12	2.333	**0.039**
Super Elfin XP White/Accent Coral	Impatiens	2.250	0.770	6.574	2.333	0.178
Super Elfin XP White/Super Elfin Ruby	Impatiens	1.778	0.402	7.863	2.333	0.634
Super Sonic Sweet Cherry/Sonic Light Pink	NG Impatiens	3.000	0.703	12.81	2.334	0.178
Sonic Light Pink/Divine White	NG Impatiens	2.500	0.565	11.06	2.334	0.317
Super Sonic Sweet Cherry/Divine White	NG Impatiens	1.200	0.409	3.521	2.334	0.916
Yellow/Matrix Purple	Pansy	6.000	1.375	26.19	2.542	**0.010**
White Blotch/Matrix Purple	Pansy	5.000	1.122	22.28	2.542	**0.029**
Mix/Matrix Purple	Pansy	5.000	1.122	22.28	2.542	**0.029**
Yellow/Mix	Pansy	1.200	0.525	2.741	2.542	0.941
Yellow/White Blotch	Pansy	1.200	0.525	2.741	2.542	0.941
White Blotch/Mix	Pansy	1.000	0.422	2.370	2.542	1.000
Easy Wave Pink/Easy Wave Neon Rose	Petunia	6.250	1.137	34.351	2.708	**0.028**
Easy Wave Pink/Easy Wave Burgundy Star	Petunia	4.167	0.989	17.560	2.708	0.053
Easy Wave Pink/Dreams Coral Morn	Petunia	2.778	0.812	9.504	2.708	0.154
Easy Wave Neon Rose/Easy Wave Berry Velour	Petunia	2.500	0.385	16.254	2.708	0.664
Easy Wave Pink/Easy Wave Berry Velour	Petunia	2.500	0.765	8.168	2.708	0.213
Easy Wave Neon Rose/Dreams Coral Morn	Petunia	2.250	0.336	15.066	2.708	0.768
Easy Wave Burg. Star/Easy Wave Berry Vel.	Petunia	1.667	0.325	8.541	2.708	0.912
Easy Wave Neon Rose/Easy Wave Burg. Star	Petunia	1.500	0.195	11.566	2.708	0.982
Easy Wave Burg. Star/Dreams Coral Morn	Petunia	1.500	0.283	7.950	2.708	0.963
Easy Wave Berry Vel./Dreams Coral Morn	Petunia	1.111	0.260	4.755	2.708	1.000

**Fig. 7.**
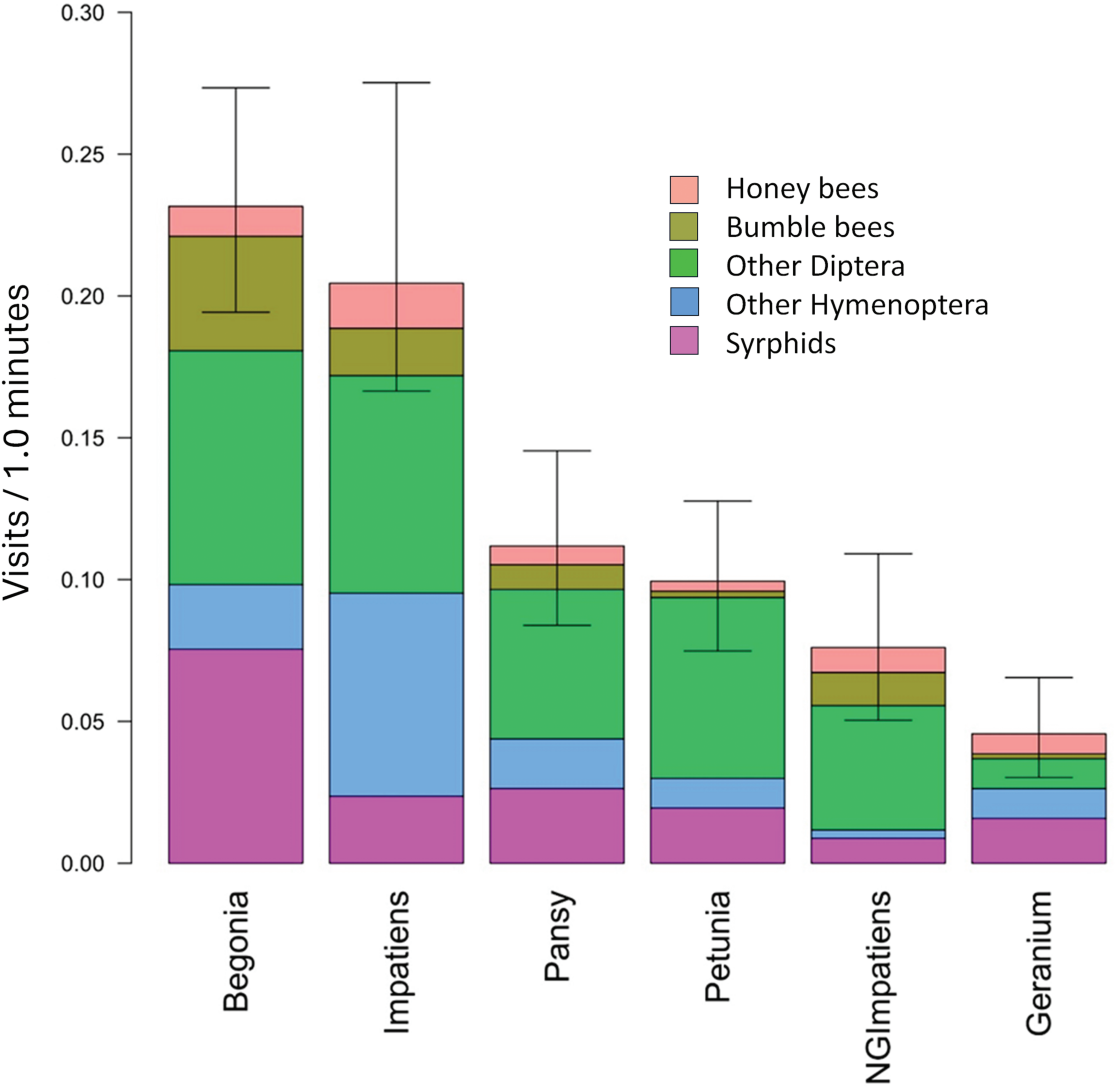
In 2018, a comparison of pollinator visits to begonia, petunia, geranium, pansy, impatiens, and New Guinea impatiens, with cultivars of each as replicates. Each bar is subdivided into visits per minute for honey bees, bumble bees, Other Diptera, Other Hymenoptera, and syrphids. Error bars at the top of each combined bar are 95% confidence limits about the mean for all 5 groups of pollinators combined. Cultivars of each flower type used as replicates are shown in Fig. 6.

In 2018, when cultivars of each annual flower are considered as replicates of that flower type, begonia and impatiens had the most total pollinator visits, with mean ± SD visits of 0.23 ± 0.03/min and 0.20 ± 0.043/min, respectively; significantly more than pansy, petunia, NG impatiens and geranium (*F* = 4.37, *df* = 5,22, *P* = 0.006, Fig. 7). Mean ± SD visits/min were 0.12 ± 0.03 for pansy, 0.10 ± 0.021 for petunia, 0.08 ± 0.03 for NG impatiens, and 0.05 ± 0.01 for geranium, with no significant differences among them (Fig. 7). As in 2017, mean bumble visits to begonia (0.04 ± 0.007 visits/min) again had more than 2-fold the number of bumble bee visits to any other type of annual.

**Fig. 8.**
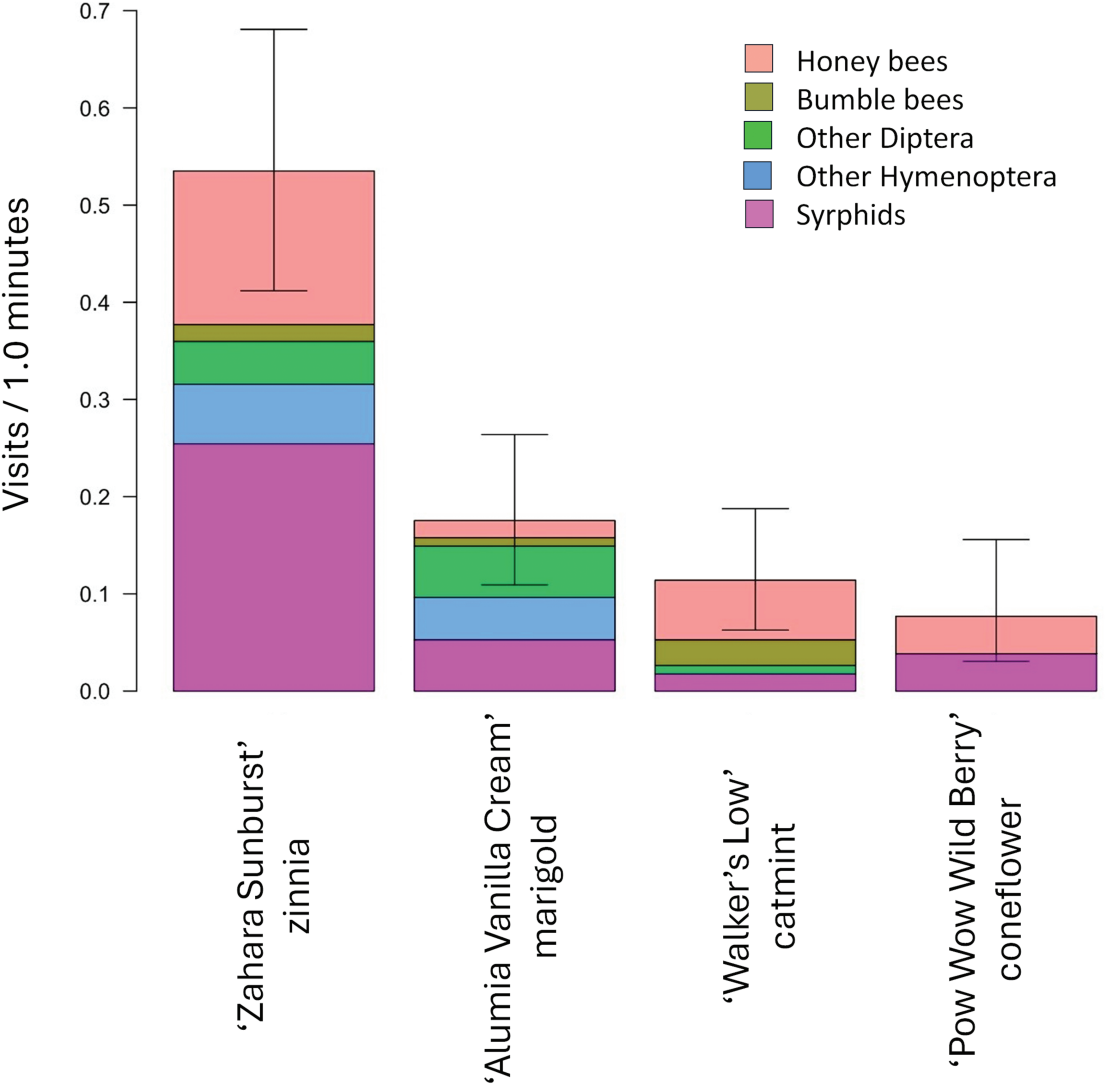
In 2018, results for pollinator-attractive benchmark plants. Data are the mean seasonal visitation rates to zinnia ‘Zahara Sunburst’, marigold ‘Alumia Vanilla Cream’, catmint ‘Walker’s Low’, and coneflower ‘Pow Wow Wild Berry’. The bar above each cultivar is subdivided into visits per minute for honey bees, bumble bees, syrphids, other Diptera, and other Hymenoptera. The error bar at the top of each combined bar is for mean visits per minute for all 5 groups of pollinators combined, with 95% confidence limits shown about the mean.

Total pollinator visits to 2 annuals and 2 perennial benchmark plants in 2018 were 0.53 ± 0.11/min for zinnia ‘Sahara Sunburst’, 0.18 ± 0.06 for marigold ‘Alumia Vanilla Cream’, 0.11 for coneflower, 0.12 ± 0.05 for catmint ‘Walker’s Low’ and 0.08 ± 0.05 for coneflower ‘Pow Wow Wild Berry’ (Fig. 8). The ‘Pow Wow Wild Berry’ plants did not grow well and produced few flowers.

### Differences in the Composition of Pollinator Visitors and Annual Flower Quality in 2017 and 2018 Field Experiments

While the total number of pollinators that visited all annual flowers and benchmark plants was similar in 2017 (*n* = 551) and 2018 (*n* = 435), the number of visits by Other Diptera doubled in 2018 compared with 2017 (Figs. 4 and 7). Another difference is the increase in Other Hymenoptera in 2018, which consists of 71% solitary bees. Other Hymenoptera visits increased for all 6 types of annuals, with an average of a 5-fold increase from 2017 to 2018. The greatest number of Other Hymenoptera visits was to impatiens in 2018, which accounted for half of Other Hymenoptera visits to all 6 annuals (Fig. 7). Total visits by honey bees and bumble bees were similar in 2017 and 2018.

Visits by honey bees, bumble bees, Other Diptera, Other Hymenoptera, and syrphids to each of the 4 benchmark plants in 2018 also changed in comparison to 2017. The biggest differences were changes in visits by syrphids, which was 3-fold greater in 2017, and in visits by Other Diptera, which was 4-fold greater in 2017. Pollinator visits to the 2 benchmark annuals, zinnia ‘Sahara Sunburst’ and marigold ‘Alumia Vanilla Cream’, were greater in 2017 (0.91 ± 0.14/min and 0.63 ± 0.11/min, respectively) than in 2018 (0.53 ± 0.11/min and 0.18 ± 0.06/min)

One unusually large difference was the 5-fold increase in visits to all cultivars of impatiens in 2018 compared with 2017, while pollinator visits to the other 5 types of annual flowers only varied ± 30% over the years. This difference is likely due to the impatiens not being shaded in 2017, which caused a reduction in flower production and stunting of growth. Shading screens placed over impatiens in 2018 resulted in more flowers, healthier plants, and apparently a large increase in pollinator visits.

Outside of the large increase in pollinator visits to impatiens because the plants were much healthier in 2018, the other differences from year to year are not unusual. Previous studies have reported fluctuation in pollinator visits from year to year due to changes in precipitation and increases or decreases in pollinator population densities for other reasons (Lázaro et al. 2010, Aldercotte et al. 2022).

## Discussion

This is the first experimental evaluation of pollinator visitation to cultivars of the most popular annual flowers sold in the United States and Europe. Overall, pollinator visitation rates to cultivars of the 6 top-selling annual flowers in our plots were low when compared with visits to our benchmark annuals, zinnia ‘Zahara Sunburst’ and marigold ‘Alumia Vanilla Cream’. Pollinator visits to the most visited annual cultivars we tested (begonia ‘Cocktail Bandy, begonia ‘Bada Boom Scarlet’, and impatiens ‘Accent Coral’ in 2018) were still 72% and 0.36% less than the number of visits to zinnia ‘Zahara Sunburst’ in 2017 and 2018, respectively. Cultivars of petunia, geranium, pansy, and New Guinea impatiens had even fewer visitors, averaging 10%–20% of that of zinnia ‘Zahara Sunburst’ over both years.

Considering the small number of pollinator visits for most cultivars of popular annuals compared with zinnia ‘Zahara Sunburst’ and marigold ‘Alumia Vanilla Cream’, it is helpful to compare pollinator visits to those 2 benchmark plants in our study with the same cultivars in other studies, to make sure enough pollinators were present to adequately evaluate the popular annuals in our plots. Browning et al. (2023) observed and collected flower visitors to 6 cultivars of marigold in replicated field plots in an agricultural setting located 2.0 km away from our research site. Their site was bordered by a horse pasture with clover and fallow agricultural fields. In that setting, Browning et al. (2023) collected 5-fold more wild bees (bee species other *Apis mellifera* and *Bombus impatiens*) than we did, while we had 0.2/min more Other Diptera, which were almost completely absent in their study. Compared with our marigold ‘Alumia Vanilla Cream’ that received 0.63 ± 0.11/min and 0.18 ± 0.06/min visits for all pollinators combined in 2017 and 2018, respectively, 5 of 8 cultivars of marigold observed by Browning et al. (2023) had a similar visitation rate by all pollinators combined (0.20–0.68/min), while 3 cultivars had higher visitation rates (0.80–1.41/min).

Erickson et al. (2020) evaluated the same cultivar of zinnia that we did and 2 cultivars of marigolds from the same breeding line as our marigold ‘Alumia Vanilla Cream’, with similar results. Erickson et al. (2020) observed visits for all pollinators combined of 0.22/min for ‘Zahara Sunburst’ plants and 0.11–0.17/min for 3 different ‘Alumia’ cultivars, while we observed 0.91 ± 0.14/min for ‘Zahara Sunburst’ and 0.63 ± 0.11/min for ‘Alumia Vanilla Cream’ in 2017, and 0.53 ± 0.11/min and 0.18 ± 0.06/min, respectively in 2018. The greater number of pollinator visits to our marigolds compared with Erickson et al. (2020) can be explained by the greater number of syrphids and Other Diptera we observed. If we compare visits by all bees, Erickson et al. (2020) observed 0.28/min for zinnia ‘Zahara Sunburst’, and 0.13–0.25/min for 3 cultivars of ‘Alumia’ marigold, while we observed 0.09/min and 0.23/min for ‘Zahara Sunburst’, and 0.12/min and 0.07/min for ‘Alumia Vanilla Cream’. One difference between our 2 studies is that Erickson et al. (2020) defined a pollinator visit as “an insect observed to be actively collecting pollen and/or nectar or coming in contact with the anthers or stigma of the plant”. We collected and later pinned and identified all flower visitors while Erickson et al. (2020) trained observers to identify pollinators in the field.

Our results from benchmark plants and comparisons to Erickson et al. (2020) and Browning et al. (2023) indicate that the low visitation rates to popular annual flowers that we observed are better explained by poor attraction to pollinators rather than by a low density of pollinators in the test area. The placement of managed honey bee and bumble bee colonies near our research plots to make sure they would be well represented did not increase the proportion of honey bee and bumble bee visits compared when compared with proportions observed by Erickson et al. (2020). In a landmark study in the United Kingdom, pollinator visits to flowers in urban, farm, and nature preserve habitats were compared (Baldock et al. 2015). The number of bumble bees, honey bees, wild bees, and syrphid visitors did not significantly vary among these habitats. The relative proportions of these 4 groups of visitors in farm habitat were syrphids, 46.3%; bumble bees, 20.6%; honey bees, 13.5%; and solitary bees, 3.9%. In our research plots located in an agricultural setting, the relative proportions of the same 4 groups were syrphids, 33.2%; bumble bees, 17.7%; honey bees, 36.7%; and solitary bees, 12.3%. Although the proportion of honey bee visitors was almost triple that observed by Baldock et al. (2015), it did not appear to inhibit visits by wild bees or bumble bees.

Sampling pollinators using modified vacuums allows accurate identification of all visitors and gives similar results to sampling pollinators by observation-only, when the 2 methods are compared (Tuell et al. 2014). However, comparing vacuum sampling with observation sampling in the same field plots over the same period does not account for visitors removed from the field plots who would have visited other flowers if they were not collected. When vacuum sampling was used in replicated field plots of 43 US native perennials, Tuell et al. (2014) observed the number of wild bees to increase as the season progressed from early to mid to late, suggesting that the removal of visitors by vacuum sampling has a small impact on visitation rates when compared with other factors.

Our results confirm previous observations of pollinator visits to annual flowers in the United Kingdom. Although none of the top 7 annual flowers (by sales volume) were sampled, a low rate of pollinator visitation to most annual flowers was reported in 2 different surveys of garden center flowers (Garbuzov et al. 2017, Rollings and Goulson 2019). In both surveys, annuals and perennials were ranked (1–111 or 1–79) based on the number of pollinator visits, with a ranking of one being the most visited. In both surveys, only 5 of the top-50 rankings were annuals (*Borago officinalis*, *Echium vulgare* ‘Blue Bedder’, *Calendula offinialis*, *Phacelia tanacetifolia*, and *Salvia viridis*), none of which are among the top 20 most popular annuals in the United States.

In our study, syrphids were the second-most abundant group of pollinators visiting our annual flowers, accounting for 24.7% of all pollinators, trailing only honey bees at 27.2%. Although known primarily as a beneficial insect because their larvae are predators, adults are also beneficial as pollinators. Jauker and Wolters (2008) observed a 15%–25% increase in rape seed yield due to pollination by syrphids and suggested their importance may increase when other pollinators are scarce. Syrphids also help pollinate fruit and vegetables in gardens (Dunn et al. 2020) and beds of wildflowers (Vance et al. 2004, Fontaine et al. 2006). The larvae of syrphid flies are important predators of 2 common plant pests: aphids and psyllids (Tenhumberg and Poehling 1995, Irvin et al. 2021). Providing nectar and pollen for adult syrphid flies encourages their presence in the garden and may enhance the biological control provided by their larvae (Nelson et al. 2012, Gontijo et al. 2013, Pineda and Marcos-Garcia 2013).

Our research has three critical limitations. First, because 25 cultivars of the 6 most popular annuals in our study have not been previously evaluated for visitation by pollinators, we needed better comparisons with cultivars of annuals that have been evaluated. Although our evaluation of replicated plots of a cultivar of marigold and zinnia as benchmark plants was helpful, it would have been better to include 4 or more annual flower benchmark plants within our blocks of popular annuals for comparison. A second limitation was evaluating annual flowers in large (18.9 l) nursery containers instead of planting them in the field. Overhead irrigation and supplemental hand-watering avoided most drought stress, but planting them in the field would provide more consistent soil moisture. Finally, shade-loving plants like impatiens cannot be evaluated in full sun, like we did in the first year of this study.

The large variation in pollinator visits to popular annuals means that plant breeders could select cultivars that attract pollinators. Our research and others like Erickson et al. (2020) and Browning et al. (2023), provide a metric that plant breeders can use: pollinator visits/plant/unit of time, if observers are trained to identify pollinators, or if they collect pollinators for identification. Including benchmark annuals within the experimental design, like zinnia ‘Zahara Sunburst’ or ‘Alumia Vanilla Cream’ marigold, would also be helpful. Counting pollinators when making other plant breeding observations is feasible, and the results could be used to support the claims of ‘pollinator-friendly’ flower types which are being promoted by plant breeders and flower marketing cooperatives.
